# Effects of Non-surgical Periodontal Therapy on Saliva and Gingival Crevicular Fluid Levels of Chemerin in Periodontitis Subjects With and Without Type 2 Diabetes Mellitus

**DOI:** 10.7759/cureus.33388

**Published:** 2023-01-05

**Authors:** Gomathi GD, Gopalakrishnan S, Uma Sudhakar, Anusha Raghavan, Keerthi V Narayan

**Affiliations:** 1 Periodontics, Priyadarshini Dental College & Hospital, Chennai, IND; 2 Periodontology, Thai Moogambigai Dental College and Hospital, Dr MGR Educational and Research Institute, Chennai, IND; 3 Periodontics, Thai Moogambigai Dental College and Hospital, Chennai, IND; 4 Public Health Dentistry, Ragas Dental College and Hospital, Chennai, IND; 5 Oral and Maxillofacial Pathology, Dr MGR Educational and Research Institute, Chennai, IND

**Keywords:** type 2 diabetes mellites, scaling and root planing, unstimulated saliva, periodontitis, gingival crevicular fluid (gcf), chemerin

## Abstract

Background

Evidence had shown a bi-directional link between diabetes mellitus and periodontitis. Chemerin, an adipose tissue-specific adipokine plays a significant role in adipocyte initiation and differentiation that directly influences glucose metabolism, lipid metabolism, and inflammatory mediators. Non-surgical periodontal therapy (NSPT) for patients with periodontitis and diabetes mellitus improves the periodontal condition and regulates glycemic level.

Aims and objectives

To assess the impact of chemerin on periodontal disease and diabetes mellitus pathogenesis and to analyze the impact of NSPT on saliva and gingival crevicular fluid (GCF) chemerin levels in patients with periodontitis with and without type 2 diabetes mellitus (T2DM).

Materials and methods

A total of 60 patients were divided into four groups: Group I: Systemically and periodontally healthy subjects (n=15), Group II: Systemically healthy subjects with periodontitis (n=15), Group III: Subjects with periodontitis and T2DM (n=15), Group IV: Periodontally healthy subjects with T2DM (n=15). Indices and parameters like plaque index (PI), gingival index (GI), periodontal probing depth (PPD), and clinical attachment level (CAL) were assessed at baseline in all four groups and six weeks after NSPT in Group II and Group III. A glycated hemoglobin (HbA1c) test was taken to assess the patient's blood glucose level. Fasting blood sugar (FBS) level was taken at baseline in all the groups and six weeks after NSPT in Group II and Group III subjects. Saliva and GCF chemerin levels were assessed at baseline in all four groups and six weeks after NSPT in Group II and Group III subjects.

Results

A statistically significant difference was observed in comparing chemerin levels at baseline with all four groups (p < 0.001). After NSPT, there was a reduction in clinical parameters, FBS, and chemerin levels in Group II and Group III. A positive correlation was observed between salivary chemerin and FBS in Group II, GCF chemerin, PI, and FBS in Group II, and PPD and FBS in Group III. A negative correlation was observed between salivary chemerin and all parameters in Group II and between salivary chemerin and GCF chemerin in Group III.

Conclusion

Based on the observed relationship between chemerin and the parameters, their utility as a dual biomarker for diagnosis and prognosis in periodontal disease seems promising. However, further studies with a larger sample size on the role of chemerin in health and various states of diseases are required to substantiate the result of the study.

## Introduction

Periodontitis is a disease of inflammatory origin characterized by gingival enlargement, bleeding on probing, loss of alveolar bone, and anchorage between the tooth and periodontium or the supporting structures in its anatomical and functional position. It is often described as a state of hard tissue imbalance between the anabolic and catabolic processes resulting in the loss of alveolar bone and supporting hard structures [1]. Diabetes, a systemic disorder with significant periodontal disease manifestation, is also viewed as an inflammatory condition and its development is preceded by a low-grade systemic inflammation [2] with elevated plasma concentrations of pro-inflammatory markers such as C-reactive proteins (CRP), cytokines (interleukin-1β, interleukin-6, tumor necrosis factor-α (alpha), and prostanoids (prostaglandin E2) [3].

Periodontitis, the sixth most common and frequently manifested oral complication in diabetic patients has been directly correlated with the levels of glycemic control [4]. Several literature studies had shown evidence that disease progression and clinicopathological manifestation in diabetes and periodontitis were observed following mild inflammatory symptoms with increased levels of pro-inflammatory markers in plasma, saliva, and gingival crevicular fluid (GCF) [4,5]. Studies on pro-inflammatory markers had also demonstrated a substantial role of cytokine levels in diabetes and periodontal disease pathogenesis. Among these markers, adipokines such as adiponectin, resistin, and chemerin, a group of cytokines have been of recent interest owing to their significant effect on insulin sensitivity and inflammatory process [6].

Chemerin, an adipose tissue-specific adipokine first identified in 1997, which is encoded by RARRES2 and secreted as a 143-amino acid precursor (prochemerin) with low biological activity [7] plays an important part in adipocyte development, initiation, and differentiation through a specific receptor chemokine-like receptor 1 (CMKLR1), and influences the glucose pathway, lipid metabolism, inflammation levels, chemotaxis of immature dendritic cells [8], and integration of macrophage-phagocytic activity to extracellular matrix proteins and adhesion molecules. Chemerin through its integrated mechanism on extracellular matrix proteins with cell-adhesion molecules aids in the union of macrophages to tissue endothelium thus exposing the defense cells and enhancing inflammatory activity [9]. The removal of subgingival plaque and calculus constitutes the cornerstone of periodontal therapy. Mechanical therapy consisting of scaling and root planing (SRP) is the gold standard for periodontal therapy. The efficacy of SRP as a part of non-surgical periodontal disease management is established through several longitudinal studies [10]. The present study was carried out to assess the impact of chemerin on the pathogenesis of periodontal disease and diabetes mellitus and to evaluate the role of non-surgical periodontal therapy (NSPT) on GCF and salivary chemerin levels in periodontitis patients with and without type 2 diabetes mellitus.

## Materials and methods

This randomized controlled trial was conducted at the department of periodontics and implantology, Thai Moogambigai Dental College and Hospital, D. MGR Educational and Research Institute, Chennai. A total of 60 subjects of both genders were randomly selected from the outpatient clinic of the department of periodontics and recruited for the study based on the eligibility criteria established for each group. Subjects were divided into four groups with 15 subjects in each group as follows:

Group I: Systemically and periodontally healthy subjects

Group II: Systemically healthy subjects with generalized periodontitis

Group III: Subjects with generalized periodontitis and type 2 diabetes mellitus

Group IV: Periodontally healthy subjects with type 2 diabetes mellitus

All patients were scheduled for a sampling of GCF, saliva, and clinical parameters assessment at baseline and after six weeks. Six weeks after SRP, Group II and Group III subjects were scheduled for a re-evaluation of clinical parameters, saliva, and GCF samples. Subjects with and without periodontitis were identified and selected according to the 2017 AAP World Workshop on Classification of Periodontal and Peri-Implant Diseases and Conditions. The subjects with and without type 2 diabetes mellitus were diagnosed based on the criteria of the American Diabetes Association. Periodontitis subjects of Group II were selected belonging to Stage II and Grade B of the disease. Periodontitis subjects of Group III were selected belonging to Stage II and Grade C of the disease.

Systemically compromised patients other than those with diabetes mellitus, pregnant or lactating women, antibiotic and/or anti-inflammatory drug regimen six months before the study, history of any therapy six months before the preliminary visit, smokers or any form of tobacco chewers, alcoholics, patients with poorly controlled diabetes (HbA1c values < 8%) or any history of diabetic complications, and patients under insulin therapy.

The following clinical parameters were evaluated for the subjects: PI by Silness J and Loe H (1964), GI by Loe H and Silness J (1963), PPD (Carranza FA et al. 2013), and clinical attachment level (CAL) (Carranza FA et al. 2013).

Collection of blood samples

Blood samples of all individuals in each group were collected after eight (8) hours of fasting overnight. Fasting blood glucose or fasting blood sugar (FBS) was measured by the glucose oxidase-peroxidase (GOD-POD) method. HbA1c (hemoglobin A1c) was measured using the standard column method.

Collection of saliva

A total of 5 ml of whole unstimulated saliva was collected from the subjects in a clean sterile container and stored at -70 ºC till the time of assay.

Collection of GCF

The site with the greatest probing depth clinically evaluated using a periodontal probe was selected for GCF collection. A supra-gingival plaque was removed after air-drying the selected region without touching the marginal gingiva (extra-sulcular method) and GCF was gradually withdrawn into the collecting tube. A standardized volume of 1 µl was collected from each site with an extra-crevicular or extra-sulcular approach, using volumetric capillary pipettes calibrated from 1-5 µl standardization. The collected GCF was transferred immediately to Eppendorf tubes and stored at - 70ºC until the time of assay.

Non-surgical periodontal treatment

Non-surgical periodontal therapy by SRP was completed under local anesthesia in two visits using Gracey curettes for Group II and Group III subjects. Clinical parameters, saliva, and GCF samples were collected in Group II and Group III after six weeks to assess the changes.

Immunological assessment of chemerin

Chemerin in saliva and GCF were identified and assessed using enzyme-linked immunosorbent assay (ELISA) in Groups I and IV at baseline and Groups II and III at baseline and six weeks after SRP.

Statistical analysis

The data were analyzed using SPSS (IBM SPSS Statistics for Windows, Version 23.0, Armonk, NY: IBM Corp). Inter-group comparisons of all parameters at baseline were analyzed using one-way ANOVA, and six weeks after SRP between Group II and Group III was done using the independent t-test. Intragroup comparison of parameters in Group II and Group III was done using the paired t-test. Correlation of salivary and GCF chemerin with all parameters in Groups II and III six weeks after SRP was done using Pearson’s correlation coefficient. The significance level was fixed as 5% (α = 0.05).

## Results

On intergroup comparison of all the parameters (PI, GI, PPD, CAL, and salivary and GCF chemerin) at baseline, there was a statistically significant reduction observed in all the groups (p<0.001) (Table 1).

**Table 1:** The intergroup comparison of clinical parameters at baseline *p<.05: significant PI: plaque index, GI: gingival index PPD: periodontal probing depth, CAL: clinical attachment level, GCF: gingival crevicular fluid, FBS: fasting blood sugar

Groups	PI	GI	PPD	CAL	FBS	HbA1c	Salivary chemerin level	GCF chemerin Level
Group I	1.01±0.06	1.03±0.2	2.61±0.299	2.658±0.28	92.0±4.85	5.3±0.26	91.9±2.65	89.2±1.90
Group II	2.78±0.23	2.8±0.25	4.72± 0.39	4.38±0.62	92.3±5.76	5.54±0.29	174.06±4.81	171.4±4.68
Group III	2.69±0.40	2.9±0.28	5.6±0.52	5.60±0.50	149.8±4.76	7.46±0.42	196.1±5.14	199.1±5.2
Group IV	0.94±0.08	1.1±0.13	3.3±0.36	3.30±0.38	140.1±6.40	7.14±0.27	134.03±5.02	133.1±4.73
p-value	0.0001*	0.0012*	0.0031*	0.001*	0.001*	0.003*	0.004*	0.002*

The inter-group comparison of all parameters between Group II and Group III showed a significant difference after six weeks in Group II (P<0.001) (Table 2).

**Table 2:** Intergroup comparison of parameters six weeks after SRP in Group II and Group III *p<.05: significant PPD: periodontal probing depth, CAL: clinical attachment level, GCF: gingival crevicular fluid, FBS: fasting blood sugar, SRP: scaling and root planing

Parameters	Group II	Group III	P - value
Plaque index	1.40±0.31	1.86±0.12	0.001*
Gingival index	1.31±0.18	1.48±0.13	0.002*
PPD	3.00±0.16	2.97±0.20	0.013*
CAL	3.05±0.15	3.03±0.19	0.001*
FBS	82.8±7.15	134.8±3.44	0.001*
Salivary Chemerin	115.9±5.22	118.6±0.19	0.007*
GCF Chemerin	119.41±6.47	125.58±6.05	0.01*

The intra-group comparison of all parameters in Group II and Group III showed a significant reduction (P<0.001) after treatment (Table 3).

**Table 3:** Intragroup comparison of clinical parameters at baseline and after one month *p<.05: significant PPD: periodontal probing depth, CAL: clinical attachment level, GCF: gingival crevicular fluid, FBS: fasting blood sugar, SD: standard deviation, sig: significant

Groups	Plaque index mean ± SD	Gingival index mean ± SD	Pocket depth mean ± SD	CAL mean ± SD	FBS mean ± SD	Salivary chemerin mean ± SD	GCF chemerin mean ± SD
Group II baseline	2.78±0.24	2.83±0.26	4.72±0.40	4.38±0.65	92.3±5.76	174.06±4.81	171.4±4.68
After 6 weeks ρ Value	1.40±0.31 0.014* Sig	1.31±0.180 0.012* Sig	3.0±0.16 0.001* Sig	3.05±0.15 0.021* Sig	82.8±7.15 0.001* Sig	115.9±5.22 0.025* Sig	119.41±6.47 0.013* Sig
Group III baseline	2.69±0.41	2.95±0.29	5.60±0.54	4.36±0.82	149.8±4.76	196.16±5.14	199.11±5.2
After 6 weeks ρ Value	1.86±0.12 0.001 Sig	1.48±0.13 0.002 Sig	2.97±0.20 0.013 Sig	3.03±0.19 0.001 Sig	134.8±3.44 0.001 Sig	118.6±0.19 0.007 Sig	125.58±6.05 0.01 Sig

The salivary chemerin showed a weak negative correlation with PI (-0.214), GI (-0.180), PPD (-0.221), CAL (-0.064), and GCF chemerin (-0.155) and a weak positive correlation with FBS level (0.338) without statistical significance in Group II (Table 4).

**Table 4:** Correlation of salivary chemerin with all parameters in Group II six weeks after scaling and root planing *p<.05: significant PPD: periodontal probing depth, CAL: clinical attachment level, GCF: gingival crevicular fluid, FBS: fasting blood sugar

Parameters	Correlation	P value
Plaque Index	-0.214	0.445
Gingival Index	-0.180	0.522
PPD	-0.221	0.428
CAL	-0.064	0.821
FBS	0.338	0.217
GCF Chemerin	-0.155	0.581

While in Group III, salivary chemerin showed a positive correlation with PI (0.272), GI (0.207), PPD (0.159), CAL (0.444), and FBS level (0.4569) and a negative correlation with GCF chemerin (-0.155) without statistical significance (Table 5).

**Table 5:** Correlation of salivary chemerin with all parameters in Group III six weeks after scaling and root planing *p<.05: significant PPD: periodontal probing depth, CAL: clinical attachment level, GCF: gingival crevicular fluid, FBS: fasting blood sugar

Parameters	Correlation	P value
Plaque Index	0.272	0.326
Gingival Index	0.207	0.459
PPD	0.159	0.572
CAL	0.444	0.097
FBS	0.4569	0.0868
GCF chemerin	-0.155	0.581

GCF chemerin showed a weak positive correlation with PI (0.166) and FBS level (0.027) and a weak negative correlation with GI (-0.088), PPD (-0.092), CAL (-0.228), and salivary chemerin (-0.155) without statistical significance in Group II (Table 6).

**Table 6:** Correlation of GCF chemerin with all parameters in Group II 6 weeks after scaling and root planing *p<.05: significant PPD: periodontal probing depth, CAL: clinical attachment level, GCF: gingival crevicular fluid, FBS: fasting blood sugar

Parameters	Correlation	P value
Plaque Index	0.166	0.555
Gingival Index	-0.088	0.755
PPD	-0.092	0.744
CAL	-0.228	0.415
FBS	0.0274	0.9227
Salivary Chemerin	-0.155	0.581

And in Group III, a weak positive correlation was seen with PPD (0.630) and FBS level (0.363) and a weak negative correlation with PI (-0.364), GI (-0.097), CAL (-0.151), and salivary chemerin (-0.126) without statistical significance (Table 7).

**Table 7:** Correlation of GCF chemerin with all parameters in Group III 6 weeks after scaling and root planing *p<.05: significant PPD: periodontal probing depth, CAL: clinical attachment level, GCF: gingival crevicular fluid, FBS: fasting blood sugar

Parameters	Correlation	P value
Plaque Index	-0.364	0.182
Gingival Index	-0.097	0.732
PPD	0.630	0.012*
CAL	-0.151	0.592
FBS	0.3638	0.1825
Salivary Chemerin	-0.126	0.656

## Discussion

Inflammation causes progressive destruction of the periodontal ligament and alveolar bone, resulting in pocket formation, gingival recession, or both [11]. Diabetes has been found to be an important host risk factor in periodontal diseases in large epidemiological studies [4]. Adipose tissue is an active endocrine organ that accumulates fat substances and generates many adipokines responsible for controlling lipid metabolism and inflammatory mechanism. Currently, there is increased evidence of adipose tissue-derived substances "the adipokines" in immune-mediated inflammatory response mechanisms. Studies on various pro-inflammatory markers had shown cytokines regulated by complex signaling pathways mediate diabetes and periodontal disease pathogenesis. One such cytokine that gained attention over the years is adipokines such as adiponectin, resistin, and chemerin postulated in diabetes and periodontal diseases for their substantial effects on insulin sensitivity and inflammatory disease process respectively [6].

Chemerin, secreted from the liver and adipocytes, is an adipokine of low-grade activity triggered by serine proteases of the hemostatic or inflammatory cascades that releases chemotactic factors by cleavage of the carboxyl-terminal peptide of the molecule thus leading to recruitment of CMKLR1-positive cells such as immature dendritic cells and macrophages [12]. SRP is intended to reduce microbial load, shrink swollen and inflamed gingiva, and recondition the subgingival ecology, making it biologically compatible with optimal healing and allowing the re-attachment of the epithelium to the root surface. The effect of SRP varies due to poor compliance with the oral hygiene regimen, inadequate debridement, composition of subgingival flora, and genetic or environmental factors [13]. Re-evaluation is usually performed a few months after non-surgical periodontal therapy to give gingival tissue adequate time to heal. The healing following non-surgical therapy occurs within three months although it may continue for nine months [14]. Reevaluation after SRP is done to determine the periodontal condition and treatment outcome [15].

Intergroup comparison of the PI at baseline showed a statistically significant difference (p<0.0001). Six weeks after SRP, the PI was reduced in Group II and Group III with a statistically significant difference (p-0.001). This might be because of the higher inflammatory condition in diseased groups when compared with the healthy group. T2DM favors plaque accumulation, thereby worsening the periodontal condition. The relationship between T2DM and periodontal disease is based on the fact that periodontal diseases, on account of the reaction to the pathogenic biofilm, stimulate chronic inflammation systemically and contribute to the inflammatory burden in the host. Hence, the more severe the disease progresses, the more the increase in inflammatory components. Intergroup comparison of the Gingival Index at baseline showed a statistically significant difference (p<0.0001). Six weeks after SRP, GI scores were reduced in Group II and Group III with a statistically significant difference (p-0.002). The decrease in the score could be due to the removal of the etiologic agent, the subgingival plaque thereby reducing the inflammatory component in the periodontal tissue. SRP will control inflammation and insulin resistance [16].

The mean PPD and CAL were comparatively high in Group III and Group II than in Group IV and Group I. Six weeks after SRP, PPD, and CAL score was reduced in Group II and Group III. On comparison, the difference was found to be statistically significant for PPD (p -0.013) and CAL (p -0.017). SRP is an effective method to reduce bacterial plaque and calculus attached to the subgingival root surface. As a consequence of SRP, host tissue can better cope with remaining microorganisms, reducing the inflammation in soft tissue and producing a varying degree of closure of the periodontal pocket. A single episode of SRP resulted in probing depth reduction and gain in clinical attachment level [14]. Group II and Group III showed a significant reduction in FBS values six weeks after SRP. Results of the study suggest that, following periodontal therapy, there is a significant improvement in glycemic control. A recent study suggested that an improvement in the subject’s periodontal health after non-surgical periodontal therapy helps improve glycemic status [17]. HbA1c is considered a beneficial indicator of long-term homeostasis, reflecting an average blood glucose concentration over a period of two to three months. In the present study, HbA1c was taken at baseline to know the actual glycemic status of the subjects in all four groups.

The mean salivary and GCF chemerin was comparatively high in Group III and Group II than in Group IV & Group I. Six weeks after SRP, the salivary and GCF chemerin score was reduced in Group II and Group III. A decrease in the levels of chemerin after treatment could be mainly because of the removal of etiological factors, which reduces inflammation in the periodontal tissues. Chemerin being a pro-inflammatory marker reduces gradually with a reduction in the inflammatory component of the disease [18]. Salivary chemerin showed a weak negative correlation with PI, GI, PPD, CLA, and GCF chemerin and a weak positive correlation with FBS level without statistical significance in Group II while in Group III, it showed a positive correlation with PI, GI, PPD, CLA, and FBS level and a negative correlation with GCF chemerin without statistical significance. This indicates that chemerin is associated with all inflammatory mediators. Chemerin is derived from immune cells that respond to periodontopathic microorganisms and it seeps from GCF into the oral fluid [19]. GCF chemerin showed a weak positive correlation with PI and FBS levels and a weak negative correlation with GI, PPD, CLA level, and salivary chemerin without statistical significance in Group II and in Group III. It showed a weak positive correlation with PPD and FBS levels and a weak negative correlation with PI, GI, CLA level, and salivary chemerin without statistical significance. An increased level of chemerin in certain chronic inflammatory diseases is mainly associated with the severity of diseases rather than its mere presence.

This proves chemerin plays a role in the inflammatory process and in periodontitis, but the exact molecular mechanism of the direct cause has not yet been completely understood. Based on the findings of the present study, pro-inflammatory markers tumor necrosis factor-alpha (TNF- α) and interleukin-6 (IL-6) increased by the severity of and progression of the disease could be a possible risk associated that affects glucose metabolism either directly or indirectly. Hence, the elevation of these cytokines attributable to periodontitis could increase the risk for insulin resistance. As low-grade inflammation is involved in the pathogenesis of systemic diseases such as type 2 diabetes mellitus, the dysregulation of cytokine secretion by adipose tissues or macrophages may be critical in disease pathogenesis [20]. Non-surgical periodontal therapy not only reduces clinically evident inflammation but also has been associated with decreasing pro-inflammatory cytokines and FBS levels, indicating that periodontal diseases have systemic effects extending beyond the local periodontal environment. This study provides a definitive substantial association of salivary and GCF chemerin in patients with periodontitis and type 2 diabetes mellitus.

The observations of the present study could be significant in understanding their role in the changing dynamics of periodontal disease progression, thereby enhancing its capacity as a diagnostic and prognostic marker of disease activity. Reduction in the levels of chemerin in saliva and GCF after non-surgical periodontal therapy could prove the effect of SRP in reducing the inflammatory component during periodontitis. It can be observed that chemerin can serve as one of the potential markers for periodontitis with diabetes mellitus. However, it also raises certain issues pertaining to the clarity of its association with metabolic parameters.

## Conclusions

The results of our study revealed that salivary and GCF expression of chemerin was higher in Group III followed by Group II and Group IV and least in Group I. This discovery is further supported by studies that confirmed a direct correlation between chemerin levels in periodontitis subjects with and without type 2 diabetes mellitus. The underlying belief among those in support of this theory is that serum chemerin levels will increase with increased adiposity. This fact takes on significant implications considering the well-understood link between central obesity and insulin resistance, the two marked peculiarities of type 2 diabetes mellitus. From the evidence from previous literature and the results of the current study, it has been established that chemerin plays a role in periodontitis subjects with and without type 2 diabetes mellitus that can be used as a disease marker. Reduction in the levels of chemerin has proven the impact of non-surgical periodontal therapy on periodontitis and diabetes mellitus.
